# Effects of vessel noise on beluga (*Delphinapterus leucas*) call type use: ultrasonic communication as an adaptation to noisy environments?

**DOI:** 10.1242/bio.061783

**Published:** 2025-03-20

**Authors:** Valeria Vergara, Marie-Ana Mikus, Clément Chion, Dominic Lagrois, Marianne Marcoux, Robert Michaud

**Affiliations:** ^1^ Cetacean Conservation Research Program, Raincoast Conservation Foundation, PO Box 2429, Sidney, BC V8L 3Y3, Canada; ^2^ Département des Sciences Naturelles, Université du Québec en Outaouais, Ripon, QC J0V 1V0, Canada; ^3^ Freshwater Institute, Fisheries and Oceans Canada, 501 University Cres., Winnipeg, MB R3T 2N6, Canada; ^4^ Group for Research and Education on Marine Mammals, 108 Rue de la cale sèche, Tadoussac, QC G0T 2A0, Canada

**Keywords:** Ultrasonic calls, High-frequency burst pulse calls, Beluga whales, Acoustic adaptations, Vessel noise, Underwater noise

## Abstract

Animal vocalizations can evolve structural features as long-term adaptations to noisy environments. Using such signals, cetaceans could mitigate masking from vessel noise. This study investigates whether beluga whales (*Delphinapterus leucas*) use ultrasonic high-frequency burst pulse (HFBP) calls to communicate in noisy conditions. We identified HFBP calls in three populations: St Lawrence Estuary, Eastern High Arctic-Baffin Bay, and Western Hudson Bay. Focusing on the industrialized St Lawrence, we investigated the effects of vessel noise on HFBP call rates compared to other call types. Ultrasonic calls, spanning a bandwidth of 36.4±6.5 to 144 kHz (Nyquist frequency), comprised 13% of the St Lawrence beluga repertoire (*n*=25,435). Noise events (*n*=21) were defined as periods when at least one vessel was visible within 2 km of the hydrophone while belugas were within 500 m. Sound pressure levels were measured before, during, and after exposure. Generalized linear mixed models revealed consistent HFBP call rates before, during, and after vessel noise exposure, while contact calls and other call types declined during exposure (*n*=4528). These findings suggest that ultrasonic signals that evolved in the Arctic – where ice-associated noise may have created a need for high-frequency communication – remain a viable communication channel in vessel noise, allowing belugas to exploit these signals to maintain communication. Understanding how belugas use signals in noisy environments can inform conservation strategies for noise-impacted marine mammals.

## Teaser

**Summary:** This study shows that belugas continue using ultrasonic calls during vessel noise, while other call types decline, suggesting belugas exploit these signals to communicate in noisy environments, despite their limitations.

## INTRODUCTION

Animal vocal signals exhibit adaptations to ambient noise that fall into two distinct categories: structural features shaped by long-term evolutionary pressures in noisy environments, and short-term vocal modifications that indicate vocal flexibility in response to noise (Brumm and Slabbekoorn, 2005). Beluga whales (*Delphinapterus leucas*), like other cetaceans (see Tyack and Janik, 2013 for review), are known to use the latter strategy to compensate for noise (e.g. Halliday et al., 2019; Lesage et al., 1999; Scheifele et al., 2005). While these short-term vocal modifications are well documented, whether belugas also rely on vocalizations with evolved structural features that facilitate communication in noisy environments (indicating the first type of adaptation) remains an open question. Certain evolved features of beluga signals are thought to be well-suited to the noisy and reverberant acoustic environment of the Arctic (Vergara et al., 2021; Vergara and Mikus, 2019). Additionally, variation in contact call parameters among beluga populations may reflect more recent adjustments to differing levels of anthropogenic noise (Booy et al., 2023). However, these hypotheses remain largely unexplored.

Belugas are among the most vocal cetaceans. Like other odontocetes, they use echolocation clicks for navigation and foraging, as well as a variety of communication calls, including narrow band whistles and chirps, broadband pulsed signals that sound like screams, squawks, creaks, or buzzes, (Belikov and Bel'kovich, 2006, 2007, 2008; Chmelnitsky and Ferguson, 2012; Fish and Mowbray, 1962; Sjare and Smith, 1986) and mixed calls, which include both signal types in the same vocalization (Karlsen et al., 2002; Vergara and Barrett-Lennard, 2008). The most extensively studied communication calls are contact calls (CCs), which are broadband (200 Hz–144 kHz), long-duration (typically >1 s) pulsed signals used for group cohesion, during isolation events (Mishima et al., 2015; Morisaka et al., 2013; Panova et al., 2017; Panova and Agafonov, 2023; Vergara et al., 2010; Vergara and Mikus, 2019) and for mother–calf contact (Ames and Vergara, 2020; Vergara and Barrett-Lennard, 2008). CCs, especially their mixed variants (referred to as complex CCs), may convey individual or familial identity (Panova et al., 2017; Vergara and Mikus, 2019).

Beluga whales also produce ultrasonic burst pulses with no acoustic energy below 20 kHz, a frequency that exceeds the human hearing range (20 Hz to 20 kHz). This portion of their vocal repertoire has remained largely understudied, as many past studies used recording equipment with low sampling rates, limiting documentation of vocal types to relatively low frequency elements (up to 24 kHz). Only one captive study documented some of the ultrasonic repertoire of this species, but it was still bandlimited to 48 kHz (Kelley, 2010). Yet discrete sequences of ultrasonic burst pulses with very brief inter-pulse intervals have been documented in other odontocetes, such as Hawaiian spinner dolphin (*Stenella longirostris*), Atlantic spotted dolphins (*Stenella frontalis*, Lammers et al., 2003), Heaviside's dolphin (*Cephalorynchus heavisidii*, Martin et al., 2019), and narwhals (*Monodon monoceros*, Walmsley et al., 2020). These studies indicate that such ultrasonic burst pulse sounds, labelled herein as high-frequency burst pulsed (HFBP) calls, serve a communication function. These calls are described as discrete sequences of clicks with high repetition rate that is consistent throughout the signal, in contrast to echolocation buzzes, which are characterized by rapid increases in the click repetition rate of an actively echolocating individual during the final stages of prey capture (Martin et al., 2019).

Given their ultrasonic nature, HFBP calls may be less susceptible to the masking effects of vessel noise, which presents a significant threat to marine mammals. Acoustic masking, which occurs when noise interferes with the detection and interpretation of conspecific signals (Clark et al., 2009; Erbe et al., 2016), is one of the most pervasive effects. Masking is influenced by factors such as the noise level, the source level (or loudness) of the vocalization, the hearing sensitivity of the listener and the frequency of both the noise and the signal (Brenowitz, 1982; Erbe et al., 2016; Miller, 2006; Reichmuth, 2012). The latter, i.e. the frequency of both the noise and signal, is key to this study: the extent to which the various beluga vocalizations are masked by vessel noise is largely determined by the amount of frequency overlap between the vocalization and the noise. Thus, in belugas, masking effects are particularly pronounced for signals with fundamental frequencies (the lowest frequency of a sound, which determines its pitch) that overlap with the frequencies of vessel noise (Erbe and Farmer, 2000; Gervaise et al., 2012; Lesage et al., 1999; Scheifele et al., 2005; Vergara et al., 2021). For instance, Vergara et al. (2021) found that vessel noise in the St Lawrence Estuary significantly reduces the communication space of CCs, with the soft calls of newborn calves being most affected due to their lower apparent source levels (the loudness or intensity of a signal at a nominal distance of 1 m from the source). By contrast, HFBP calls, which contain most of their acoustic energy above the frequency range of vessel noise, may offer a less vulnerable communication channel. Yet, whether belugas preferentially use ultrasonic calls that are part of their vocal repertoire in noisy conditions remains an open question.

This study investigates this question by first identifying HFBP calls in three distinct beluga populations to confirm their presence as part of this species’ repertoire. It then focuses specifically on the endangered St Lawrence Estuary (SLE) belugas (Species at Risk Act, 2017), a population exposed to intense anthropogenic noise from recreational and commercial vessel traffic in the Saguenay-St Lawrence Seaway (DFO, 2017, 2020; Gervaise et al., 2012; Lesage, 2021; Lesage et al., 2014; McQuinn et al., 2011; Simard et al., 2010). This exposure is particularly concerning in Baie Sainte-Marguerite, a critical summer habitat for females and calves (Lefebvre et al., 2012). By analyzing the relative use of ultrasonic and other call types before, during and after exposure to vessel noise in Baie Sainte-Marguerite, we assess whether belugas use ultrasonic signals – evolved in the acoustically complex and often noisy Arctic environment – as a strategy to communicate in vessel noise.

## RESULTS

### HFBP usage in three beluga populations

The proportional usage of HFBP calls in the SLE, Western Hudson Bay (WHB), and Eastern High Arctic - Baffin Bay (EHA-BB) populations is listed on Table 1. In Baie Sainte Marguerite (SLE) 13% of the vocalizations were monophonic high frequency burst pulse calls (HFBP-M) without acoustic energy below 30 kHz, a slightly higher percentage than in the other two locations, both with 9% production of HFBP-M calls (see Materials and Methods). The proportional use of high frequency burst pulse calls with a low frequency element (HFBP-B), however, was much higher in Baie Sainte-Marguerite (31%) compared to the Churchill River Estuary (3%), which is the other location where HFBP-B calls were classified and counted. Additionally, the proportional use of all ultrasonic calls (HFBP-M, HFBP-B) differed significantly between Baie Sainte-Marguerite and the Churchill River Estuary, the two populations for which all high-frequency calls were classified and counted (*χ*_2_=2006.2, d.f.=2, *P*<0.001).

**Table 1.** Proportional usage of ultrasonic calls by three beluga populations

Location (Population)	N	HFBP-M	HFBP-B	Other calls	Time analyzed
Baie Sainte-Marguerite (SLE)	25,435	13%	31%	56%	19:25:40
Churchill River Estuary (WHB)	5035	9%	3%	88%	2:05:44
Cunningham Inlet (EHA-BB)	9746	9%	Not counted*	91%	24:00:00

*HFBP-B calls in Cunningham Inlet were included with ‘other calls’.

### Noise analysis for all Baie Sainte-Marguerite events

**Fig. 1.**
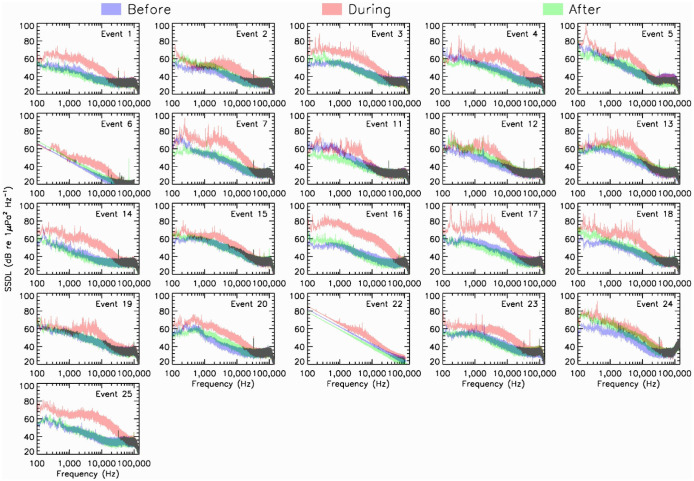
**Average SSDLs spectra (in dB re 1 μPa^2^ Hz^−1^) as functions of the frequency for before (purple), during (red), and after (green) segments of selected noise events.** All but two noise events (*n*=21) were processed from 100 to 144,000 Hz. Events 6 and 22 were processed up to 128,000 Hz due to the lower sampling rate of the icListen hydrophone.

Each 5-s segment of the before, during, and after treatments that constitute each noise event consists of 10 SSDLs spectra, each lasting 0.5 s (see Materials and Methods). PAMGuide output spectrograms were averaged (i.e. we calculated the average noise level for each 1-Hz frequency bin over 5 s) to produce a mean spectrum for each of the three 5-s segments. These are shown in Fig. 1.

**Fig. 2.**
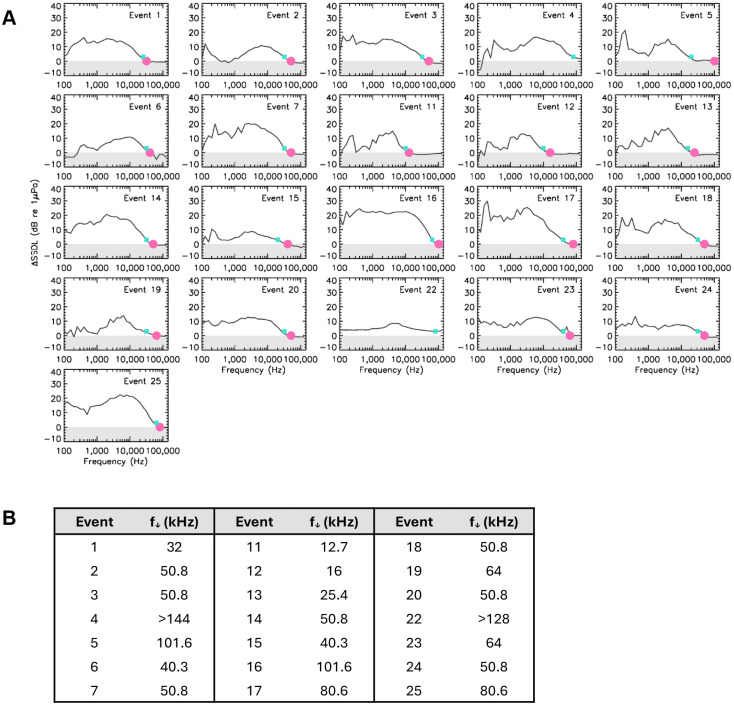
**Signal excesses per band in dB re 1 μPa (retrieved from individual panels of Fig. 4) defined as the 1/3-octave difference ΔSSDL≡SSDL (during) - <SSDL(before, after)>.** The **<>** notation on the previous equation indicates the average between the before and after spectra. The shaded area in each panel shows where the SSDL (during) signal would be statistically indiscernible from the before and after segments (i.e. ΔSSDL≤0 dB re 1 μPa). The pink dot indicates the frequency (*f*_↓_) at which the ΔSSDL curve, in black, enters the shaded area (when applicable). The turquoise square marks a lower limit for *f*_↓_ considering an expected uncertainty on hydrophone-based measurements of ±3 dB re 1 μPa (Bertschneider et al., 2014).

For smoothing considerations, spectra were then frequency-integrated into 1/3-octave bands. The signal excess per band (ΔSSDL; in units of dB re 1 μPa) was defined as the difference between the during noise levels and the average between the before and after noise levels. Results for ΔSSDL are shown in Fig. 2.

In each panel of Fig. 2, the frequency (*f_↓_*) at which the signal excess (ΔSSDL) curve falls in the gray-shaded area indicates when the vessel noise becomes acoustically undetectable. The frequencies above which the vessel's signal is no longer discernible from the ambient noise levels are listed under the figure for all selected events. Note that for Events 4 and 22, the vessel noise dominates the acoustic signal across the respective hydrophones’ bandwidth. These events correspond to recreational small vessels running at full speed between 500 m and 2 km from the hydrophone. The average value for *f_↓_* is 53.4±24.8 (1σ) kHz with 5%, 25%, 50%, 75%, and 95% percentile envelopes, respectively, at 12.7; 40.3; 50.8; 64.0; and 101.6 kHz.

### Beluga call types during Baie Sainte-Marguerite noise events

During our noise events (see Materials and Methods), we classified a total of 4528 calls, divided into 468 broadband CCs, 2198 HFBP, and 1862 other call types (which included whistles, chirps, etc.). HFBP calls were the only call type that were produced at similar rates before, during and after noise events, with no significant difference between before versus during noise (estimate=−0.294, s.e.=0.261, z=−1.129, *P*=0.5175) or after versus during noise (estimate=−0.124, s.e.=0.256, z=−0.486, *P*=0.6270).

**Fig. 3.**
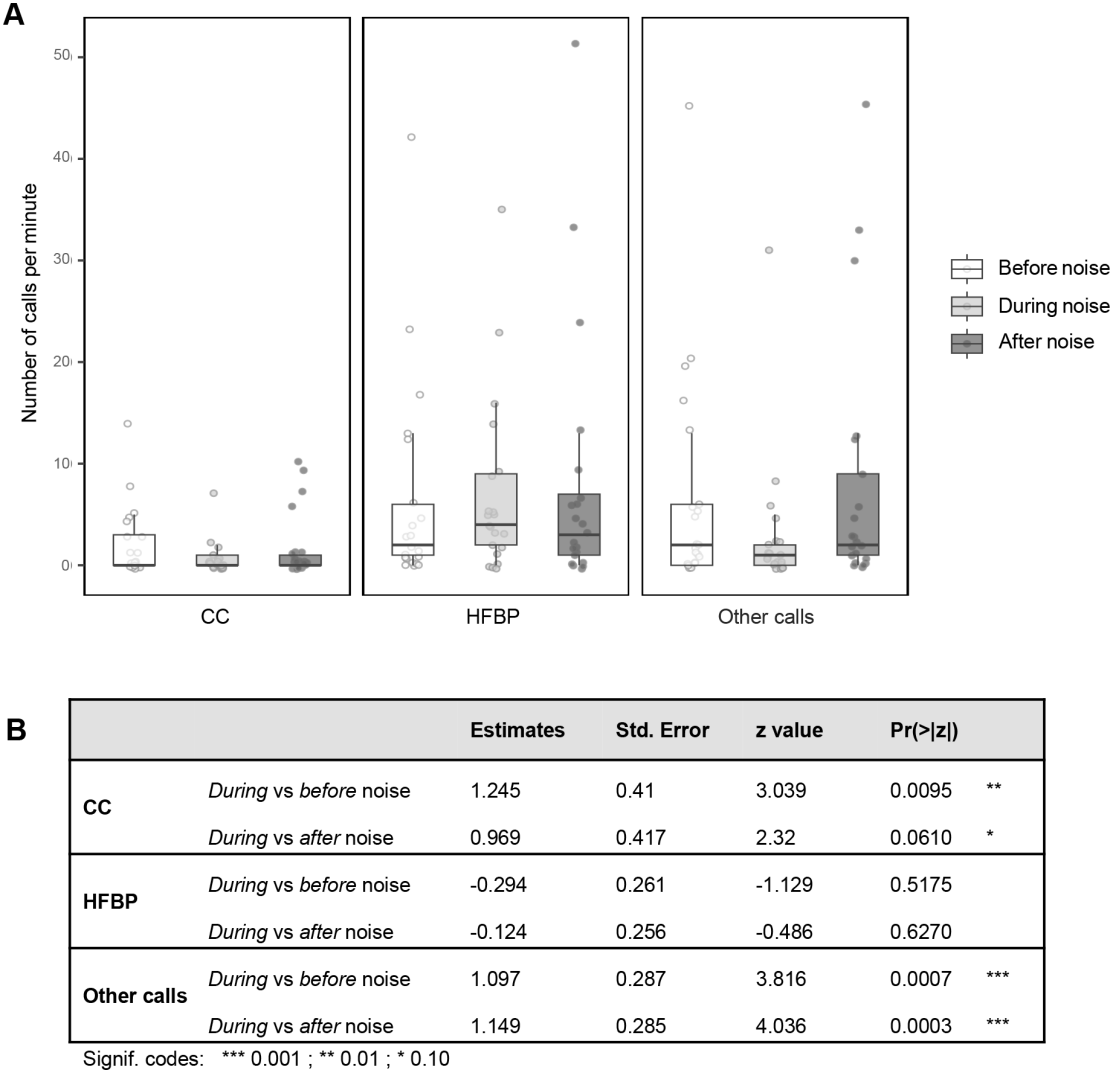
**Effects of vessel noise on beluga call rates in Baie Sainte-Marguerite.** (A) Number of calls per minute across three noise conditions: before noise, during noise, and after noise, with *n*=21 for each condition. Each panel represents a different call type: CCs, HFBP calls, and all other call types (other calls). Boxplots represent the interquartile range (IQR), with medians indicated by horizontal lines within the boxes. Whiskers extend to 1.5 times the IQR, and individual data points are displayed as grey circles. (B) Parameter estimates, standard errors, z-values, and *P*-values from generalized linear mixed models (GLMMs) with a negative binomial distribution. The models assessed the relationship between call rates (number of calls per minute) and noise conditions (before, during, after) with noise events included as random effects (with *n*=21 for each condition). Pairwise comparisons were conducted using estimated marginal means (emmeans) with Holm correction for multiple comparisons. Details of the statistical analysis can be found in the Materials and Methods.

In contrast, the CC rate was significantly higher before noise events than during noise events (estimate=1.245, s.e.=0.41, z=3.039, *P*=0.0095). After noise events, the CC rate exhibited a non-significant trend suggesting an increase (estimate=0.969, s.e.=0.417, z=2.32, *P*=0.0610). Finally, the rate of all other call types was significantly higher before noise events (estimate=1.097, s.e.=0.287, z=3.816, *P*=0.0007) and after (estimate=1.149, s.e.=0.285, z=4.036, *P*=0.0003) than during noise events (Fig. 3).

**Fig. 4.**
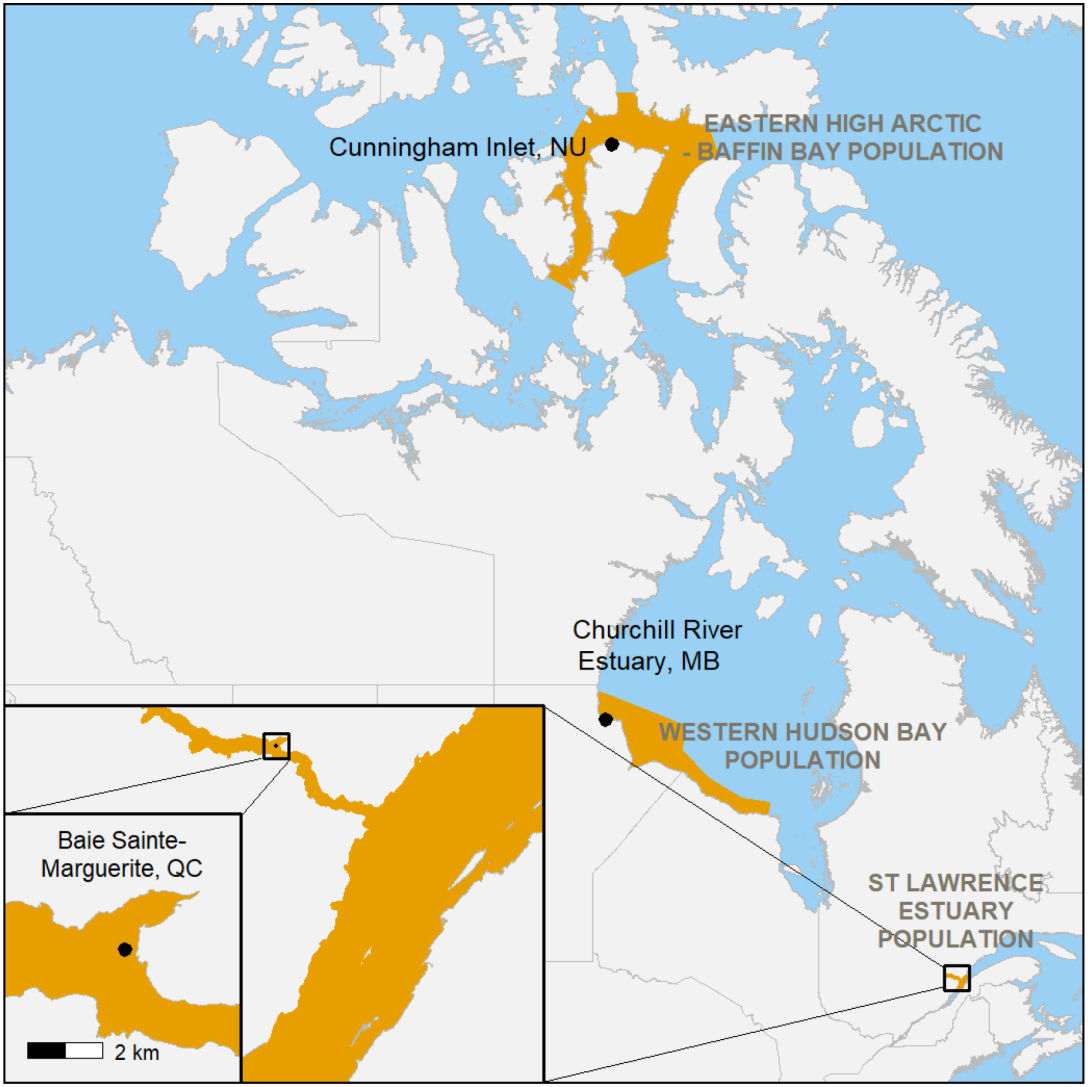
Map indicating the location of the three studied beluga whale populations and the approximate location of the hydrophones.

The *Pseudo R^2^*, which is the proportion of variance explained by the fixed effects treatment (i.e. the before, during and after treatments) and by call types (CCs, high frequency calls and other calls) was 18.2%, while the random effect event (i.e. the 21 noise events) accounted for 67.99% of the variance. This means that the factors we did not include in the model, such as herd behavior, could only explain 13.81% of the variance in the data, suggesting that most of the variability in call rates is indeed explained by our model.

## DISCUSSION

This study identified HFBP calls in all three sampled populations, suggesting that these ultrasonic calls are part of this species repertoire. The results further revealed that in the industrialized St Lawrence Estuary, ultrasonic calls were the only category produced at similar rates before, during and after noise events, indicating that the production of these high frequency calls did not seem to be affected by noise events. In contrast, the rate of CCs showed a significant drop during noise events compared to before, while the rate of all other call types was significantly higher both before and after than during noise events, hinting at an impaired ability to use non-ultrasonic calls effectively in noise. Our results show that most of the variability in call rates (84.2%) is explained by the model.

The extent to which vessel noise masks cetacean vocalizations is largely determined by the frequency overlap between the vocalization and the noise. It is now well known that shipping noise can extend to high frequencies used by some high frequency odontocetes for echolocation and communication (e.g. Hermannsen et al., 2014; Veirs et al., 2016). Yet, both HFBP-M and HFBP-B calls have median peak, center, and Q3 frequencies (see Table 2) above the frequencies that were found to contain much of the acoustic energy in vessel noise. Our results indicate that the frequency above which the vessel noise is no longer discernible from the ambient noise levels was, on average, 53.4±24.8 (1σ) kHz. It is thus unsurprising that ultrasonic call production remained unchanged during noise events.

**Table 2.** Acoustic parameters of the SLE HFBP calls analyzed in this study

Measured parameter	Description	HFBP-M calls (*n*=100)		HFBP-B calls (*n*=100)		LF component of HFBP - B calls	
		Average±s.d.	Median	Average±s.d.	Median	Average±s.d.	Median
Low freq. (kHz)	Lowest frequency of the main pulse train	36.4±6.5	37	34.5±6.7	34.7	0.8±0.79	0.53
High freq. (kHz)	Upper frequency limit of the main pulse train	144±0	144	144±0	144	11.9±6.3	12.5
Delta time (s)	Duration of the call	0.4±0.3	0.3	0.4±0.4	0.3	0.3±0.2	0.3
Delta freq. (kHz)	Total bandwidth of the call	107.6±6.5	107.0	143.2±0.8	143.5	11.1±6.5	11.7
Peak freq. (kHz)	Frequency at which the maximum power occurs within the call	55.5±16.3	49.1	33.4±28.7	43.6	1.9±1.7	1.5
Center freq. (kHz)	The frequency that divides the call into two intervals of equal energy	67.3±11.3	67.5	63.6±15.6	62.3	1.9±1.2	1.6
Q1 freq. (kHz)	Frequency that divides the call into two frequency intervals containing 25% and 75% of the energy in the call	53.7±6.8	52.8	46.3±16.4	48.8	1.3±0.9	1.2
Q3 freq. (kHz)	The frequency that divides the call into two intervals containing 75% and 25% of the energy in the selection	83.8±11.2	86.5	84.7±11.1	85.0	3.0±2.1	2.7

In contrast, the significant reduction in CCs and other beluga call types during noise events aligns with the fact that any non-ultrasonic call would exhibit a more substantial frequency overlap with vessel noise, rendering it more susceptible to masking. Attempting to communicate using these non-ultrasonic calls during noise events may be inefficient and energetically costly. In the St Lawrence Estuary, adult and subadult contact calls, as well as those emitted by newborn calves, exhibited a 57% and 53% reduction in communication range, respectively, in the presence of vessel noise (Vergara et al., 2021). An earlier evaluation of masking of wild beluga calls by St Lawrence vessel noise used a theoretical narrowband signal with an assumed central frequency of 2.5 kHz, and estimated a potential decrease in communication range from 4.5 km in natural ambient noise to 1.5 km 50% of the time and to 0.6 km 25% of the time in the busy mouth of the Saguenay Fjord (Gervaise et al., 2012). In Cook Inlet, Alaska, a beluga's communication band, assumed to extend to 16 kHz (i.e. not in the ultrasonic range), was found to experience full masking from ambient noise and underwater radiated noise emitted by a containership up to 5000 m, and partial masking up to 10,000 m and beyond (Eickmeier and Vallarta, 2023). In the same study, the 32 to 100 kHz ultrasonic echolocation band was only partially masked within a range of 2500 m from a container ship, with no masking effects observed at greater distances. This finding is particularly relevant to our study, as it highlights the differential impact of vessel noise on various frequency bands, including those in the ultrasonic range.

While using ultrasonic calls may offer a temporary solution in noisy environments to overcome the masking effects of background noise on beluga communication, it is not without its limitations. Higher frequency sounds are absorbed more rapidly as they travel through seawater. As the frequency of sound increases, the distance over which the sound energy is absorbed by seawater decreases (Tyack and Janik, 2013). This shortens the effective communication range of these signals, making it more difficult for belugas to communicate over long distances. For this reason, burst pulse signals in other odontocetes are believed to be used for communication with nearby conspecifics (Lammers et al., 2003).

In addition to limiting the spatial extent of communication, a shift toward primarily using ultrasonic calls during noise events may also influence the nature and complexity of information exchanged. The specific function of HFBP calls in belugas remains unclear but burst-pulse vocalizations in odontocetes have been associated with social interactions and are believed to have a communication function (e.g. Lammers et al., 2003; Martin et al., 2019). Since beluga communication also relies on non-ultrasonic call types, an over-reliance on HFBP calls during noisy events may constrain the richness of social information that can be conveyed. Further research is needed to determine whether ultrasonic calls serve the same communicative functions as other beluga vocalizations, and whether such a shift in call usage affects social dynamics.

Of the three sampled beluga populations the SLE showed the highest proportional usage of ultrasonic burst pulsed calls, both with and without biphonation. Given our findings that all call production outside the ultrasonic range decreases during vessel noise events and considering the pervasive underwater noise pollution in the SLE, the prevalence of HFBP call usage by this population compared to the other sampled populations could indicate an adaptive strategy. Beluga whales may use ultrasonic calls more frequently in noisy conditions compared to other call types. In less industrialized habitats, where noise levels are lower, there may be less need to rely on these short-range calls for communication, given the limitations of ultrasonic communication noted above.

Booy et al. (2023) examined geographic variation in contact calls among four Canadian beluga populations, the St Lawrence Estuary, the Eastern Beaufort Sea, the Eastern High Arctic-Baffin Bay, and the Western Hudson Bay. Their study supports our findings, showing that contact calls exhibited higher peak frequencies and duration in the SLE population, where noise pollution is highest. Conversely, contact calls recorded from the Eastern High Arctic-Baffin Bay population during their summer residency in Cunningham Inlet, characterized by exceptionally low levels of anthropogenic disturbance, exhibited the lowest average peak frequencies. The authors suggest that the differences in overall ambient noise in the summer habitats of these populations influence the acoustic attributes of beluga calls.

Similarly, the ultrasonic calls described here may have evolved as an adaptation to the highly reverberant and noisy Arctic environment. Although the Arctic can be an exceedingly quiet environment under the ice during calm weather conditions, the ice sheet itself can generate noise. It can produce transient broadband impulsive noise underwater when it cracks and fractures, and broadband frequency-modulated sounds with the opening of ice leads (e.g. Kinda et al., 2015). These geophonic signals have frequencies below 4 kHz (Cook et al., 2020; Kinda et al., 2015), which overlaps with the frequency range where the acoustic energy of vessel noise, especially from large vessels, is dominant. The fact that belugas and the closely related narwhals – both Arctic species – produce high frequency ultrasonic pulsed calls that serve a communication function (see figure 1 in Walmsley et al., 2020) suggests that this signal structure could have evolved as an adaptation that may allow these species to communicate, at least when in close range, above the frequency bands of these ice-associated noise sources.

Considering that belugas are high-frequency odontocetes, a functional hearing group (see Southall et al., 2019) capable of perceiving sound waves in the range of up to 150 kHz, with their most sensitive hearing range between 16 and 100 kHz (Mooney et al., 2018), their ability to use high frequency calls to communicate is fitting. A close alignment between the acoustic characteristics of beluga whale communication signals and the specific frequencies to which their auditory systems are sensitive makes adaptive sense.

In light of our study's findings, it is apparent that ultrasonic burst pulse calls in beluga whales may have evolved as adaptations to communicate effectively in noisy environments. However, it is important to acknowledge the limitations of our dataset, particularly the differences in recording durations and methodologies across the three populations. The recordings from the Churchill population were limited in duration compared to those from the other sites, as the data were collected during targeted 5-min sampling sessions rather than continuous monitoring. While these recordings provided valuable insights, the shorter duration and opportunistic sampling may not fully capture the range of acoustic behaviors or account for variability in call usage over time. Additionally, the focus on the St Lawrence population for the experimental component of the study further limits direct comparisons among populations. These constraints underscore the need for caution in generalizing our findings to broader behavioral patterns or environmental contexts and highlight the value of future studies with more standardized and extensive sampling efforts.

Despite these limitations, our study emphasizes the role of specialized signals that allow belugas to compensate for vessel noise by relying on such signals, although it remains unclear what energetic, efficiency, or communicative costs this compromise may impose. Our research highlights the interplay between natural acoustic environments and anthropogenic noise in shaping communication behavior. It underscores not only the adaptability of cetacean communication strategies but also the importance of considering such adaptations in conservation efforts and management strategies amid increasing anthropogenic noise in marine environments.

## MATERIALS AND METHODS

### Study populations

To establish that ultrasonic calls are used by belugas in various populations, we identified HFBP calls recorded from three sites, and estimated their rate of production: the busy, high-traffic Baie Sainte-Marguerite, within the range of the SLE population, the less industrialized Cunningham Inlet, frequented by the EHA-BB population and Churchill River Estuary, home to the WHB population, which experiences some vessel activity but at much lower levels than the SLE (Fig. 4, Table 3).

**Table 3.** Location, year, equipment type, sampling rate and method of visual observations for each study population

Population	Sampling site	Coordinates (latitude longitude)	Year	Equipment	Sampling rate	Visual observations
EHA-BB	Cunningham Inlet	74.083 −93.750	2014 2015	IcListen HF (Ocean Sonics)	256 kHz, 24 bits	From shore and from observation tower
WHB	Churchill River Estuary	58.746 −94.200	2017	IcListen HF (Ocean Sonics)	256 kHz, 24 bits	From boat
SLE	Baie Sainte-Marguerite	48.251 −69.967	2017 2018	SoundTrap 300 HF	288 kHz, 16 bits	From observation tower

Population abbreviations are as follows: EHA-BB, Eastern High Arctic-Baffin Bay; WHB, Western Hudson Bay; SLE, St Lawrence.

The latest estimate of the SLE population is 1850 individuals (DFO, 2023). Baie Sainte-Marguerite lies just 500 meters from the primary navigation route in the Saguenay Fjord. In 2017, there were 342 cargo ship transits and 105 large cruise ship transits in the Saguenay fjord, with peak activity between September and November when belugas are present in the region (Turgeon, 2019). Although precise figures are unavailable, several thousand small boats, primarily commercial tourist boats and pleasure crafts, navigate in the Saguenay between May and October. Additionally, more than 40,000 ferry transits occur annually at the mouth of the Saguenay fjord, resulting in close encounters with virtually all belugas traveling to or from Baie Sainte-Marguerite. This intense traffic makes the SLE population ideal for studying the impacts of anthropogenic noise.

In contrast, the EHA-BB and WHB populations inhabit regions with significantly lower levels of vessel traffic. The size of the EHA-BB population was estimated at 21,200 individuals based on an aerial survey conducted in 1996 (Innes et al., 2002). In 2014, when the recordings for this study took place (Table 3), no ship equipped with AIS entered the Cunningham Inlet sampling site due to its inaccessibility to large vessels, and no vessels of any kind were present in the area during the recordings used in this study.

The size of the WHB beluga population was estimated at 54,500 animals based on an aerial survey conducted in 2015 (Matthews et al., 2017). Although the port of Churchill, located within the distribution of the WHB population, is a major marine hub connecting the Canadian Arctic to Europe (Arctic Bridge, Dawson et al., 2018), the temporary closure of the port in 2017 resulted in only three AIS-equipped vessels entering the WHB sampling area during our study period. Occasional noise from whale-watching boats is present in Churchill; however, there are only two whale-watching companies operating in the area, with excursions occurring only during limited hours of the day.

Given the minimal vessel traffic in the Cunningham Inlet and Churchill River Estuary, these sites were unsuitable for assessing the effects of noise events on beluga communication. Instead, these recordings were included to demonstrate that HFBP calls are part of the beluga vocal repertoire and are produced by beluga whales across different populations. The experimental component of this study, focused on the impact of vessel noise, was therefore conducted exclusively in the St Lawrence Estuary, where vessel traffic was sufficiently frequent to provide an adequate dataset for analysis.

### Call types

**Fig. 5.**
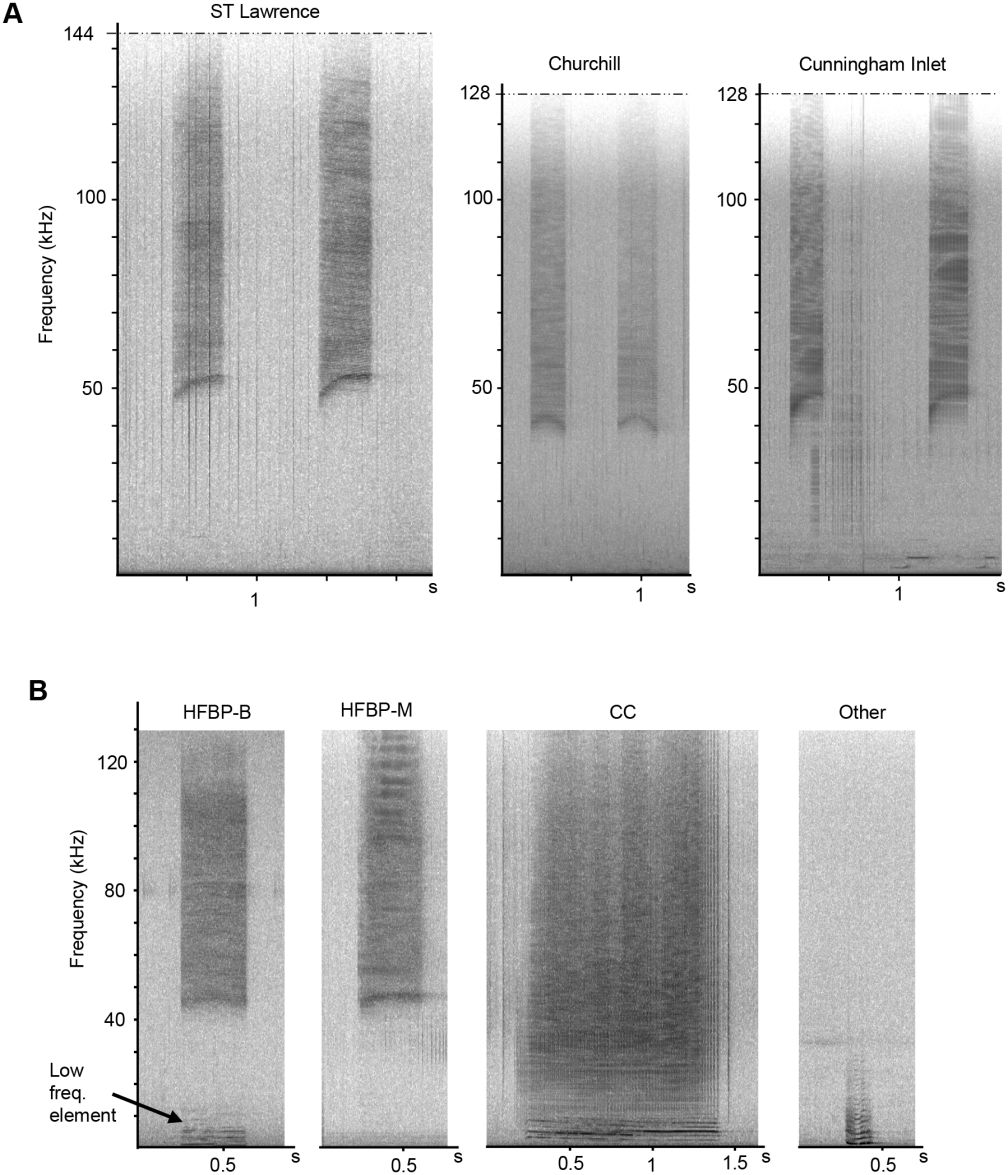
**(A) Examples of HFBP calls recorded in the three studied populations (window type=Hann, window size=1024).** (B) Spectrograms illustrating the beluga vocalization types classified for the 21 noise events: monophonic high frequency burst pulse calls with no acoustic energy below 30 kHz (HFBP-M), biphonal high-frequency burst pulse calls with a low frequency element (HFBP-B), broadband mixed contact calls (CCs) and all other call types (other).

We used Raven Pro 1.5 (Cornell Lab of Ornithology) to examine the recordings both aurally and spectrographically. We selected all HFBP calls, defined as burst pulse trains with the lowest frequency of the broadband pulses at 20 kHz or above, extending to the Nyquist cutoff (half the sampling rate, which was either 256 kHz or 288 kHz). Fig. 5A illustrates the typical structure of HFBP calls in all three populations.

To differentiate HFBP calls from the terminal echolocation buzzes used for foraging, which were not included in our analysis, we identified HFBP calls as those not immediately preceded or followed by echolocation clicks. This methodological distinction aligns well with approaches taken in previous studies, which have similarly separated burst pulses from buzz trains based on a lack of temporal association (e.g. Arranz et al., 2016, for Risso's dolphins, *Grampus griseus*; Holt et al., 2019, for Killer whales, *Orcinus orca*; Blackwell et al., 2018, for narwhals). Additionally, HFBP calls were distinguished from terminal echolocation buzzes by their frequency modulation, a characteristic not typically observed in buzzes.

HFBP calls can be biphonal, containing a low frequency tonal element and no acoustic energy between the low frequency and the high frequency components (HFBP-B), or monophonic, without a low frequency component (HFBP-M). Fig. 5B illustrates both types of ultrasonic calls (HFBP-M and HFBP-B), in addition to the other call types selected for the experimental portion of this study, which will be described in a later section. Although HFBP calls were identified for all three populations, HFBP-B calls (i.e. with a low frequency element) were counted for two of the three studied populations, SLE and WHB.

### St. Lawrence Estuary study site: Baie Sainte-Marguerite

The experimental aspect of this study took place in Baie Sainte-Marguerite (BSM), Canada, a small delta on the left bank of the Saguenay River, approximately 12 nautical miles upriver from the confluence of the Saguenay and St Lawrence. This was a natural experiment, relying on observations of naturally occurring noise events rather than the artificial introduction of standardized noise sources.

The deepest channel is in the center of the Saguenay River, at 118 m, gradually becoming shallower towards the mouth of the Sainte-Marguerite River, with a depth of 10 m. BSM's intertidal zone is separated from the opposite shore of the Saguenay River by 0.8 nautical miles. Given this relatively short distance and the steep rocky cliffs that characterize the area, the noise from any motorized vessel transiting upstream or downstream can propagate throughout the whole area, from shore to shore (Vergara et al., 2021).

### General Baie Sainte-Marguerite ambient noise recordings

A SoundTrap HF300 (Ocean Instruments, NZ) with a flat frequency response from 20 Hz-150 kHz (±3 dB), an end-to-end sensitivity of −172.7 dB re 1 V/µPa^−1^, 16-bit resolution and a sampling rate of 288 kHz was deployed at a depth of 15-20 m. The device recorded nearly continuously from 24-Jul to 18-Aug 2017, for a total of 23 days of recordings, and from 07-Jul to 17 Aug 2018, for a total of 35 days of recordings. We secured the SoundTrap to our observation platform using a sink line, anchored it in place, and fastened a buoy to keep it suspended a short distance above the riverbed. For two of our noise-event sequences (described below), we used a calibrated icListen HF hydrophone (Ocean Sonics), with a frequency response of 10–200 kHz, an end-to-end sensitivity of −170 dB re 1 V/µPa^−1^, 24-bit resolution and a sampling rate of 256 kHz.

### Baie Sainte-Marguerite visual observations

We erected a 6 m high observation tower, with a 1.5×3 m platform, during the spring tide window at the mouth of the Sainte-Marguerite River, at 48.251 N-69.967 W. The tower was surrounded by water most of the time, except for periods coinciding with peak low tides. We accessed it with a small boat and spent up to 8 h/day on this platform to document vessel activity (number and type of visible boats running or idling, and distance category from the hydrophone), in addition to presence or absence of belugas in the bay, herd radius, herd composition, number of animals in the herd, and distance from the hydrophone.

### Baie Sainte-Marguerite noise events

**Fig. 6.**
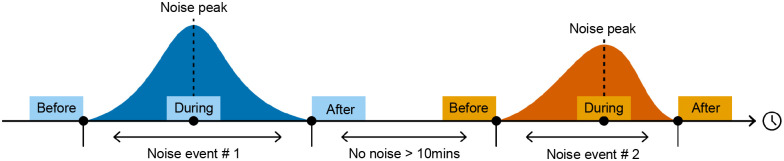
**Diagram illustrating the three treatments of the experimental design: before, during, and after audible boat noise.** Before: 5 min without vessel noise before it becomes audible. During: 5 min centered around the peak of audible boat noise, when one or more boats visible within 2 km from the hydrophone. After: 5 min without boat noise just after boat noise stops being audible (n=21 noise events).

Fig. 6 illustrates our experimental design. A noise event is defined as a visible and audible boat within 2 km of the hydrophone (the area visible from the tower, considering river bends) when the beluga herd was within 500 m from the hydrophone. We visually and aurally scanned through our BSM recordings, selecting acoustic sequences of up to 5 min in duration before a noise event (no audible noise in the recordings, no boats <2 km), during a noise event (centered on the noisiest period when one or more boats were visible within 2 km of the hydrophone) and after a noise event (noise no longer audible, no boats <2 km). For events to be considered separate, the period between them had to be longer than 10 min. A total of 25 noise events from our 2017 and 2018 recordings met those criteria. After discarding four events (event numbers 8, 9, 10 and 21) during which no calls were produced, we analyzed 21 events across 14 different days.

### SSDL measurements during noise events

We estimated sound spectral density levels (SSDLs; in units of dB re 1 μPa^2^ Hz^−1^) in each treatment (i.e. before, during and after recordings) for all 21 events, to examine the degree of overlap of HFBP calls with the acoustic energy in vessel noise. To do so, a total of 15 s of background noise without beluga vocalizations were selected for each of the 21 events (5 s before, 5 s during and 5 s after). This was achieved by visually reviewing spectrograms using Raven Sound Analysis Software (Cornell Lab of Ornithology) and selecting 10 brief periods of 0.5 s without any beluga vocalizations throughout the treatment until they added up to 5 s for each treatment.

MATLAB-supported PAMGuide (Merchant et al., 2015) was used to convert the 5-s WAV files into power spectral density plots, estimating the mean SSDL in 1-s time windows from 10 to 144,000 Hz for the SoundTrap HF300 hydrophone and from 10 to 128,000 Hz for the icListen HF device. SSDLs were extracted using the Welch method (Welch, 1967) with a Hann window (Cerna and Harvey, 2000) and a 50% overlap.

In shallow waters, the underwater topography acts as a high-pass filter and acoustic signals with frequencies below *f*_0_ cannot properly propagate. The cut-off frequency *f*_0_ is given by:

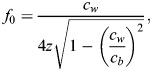
where *z*=20 m is the hydrophone's depth, and *c_w_* and *c_b_* are, respectively, the speeds of sound in the water and the sub-terrain. For BSM, *c_w_*/*c_b_*=0.95 (Loring and Nota, 1973) indicating that the signal measured at frequencies below approximately 75 Hz in during events may not be necessarily associated to the passing ship but rather to very localized noise close to the hydrophones (e.g. flow noise attributed to tidal currents). Only the acoustic signal above 100 Hz was considered in the analysis to follow.

### Beluga call types during Baie Sainte-Marguerite noise events

We selected all the beluga vocalizations identified during the 21 events and classified them as CCs, HFBP calls or other (all the other types of calls not fitting into the CC or HFBP categories, which were mostly whistles and short broadband burst pulse signals), using Raven Sound Analysis Software (Cornell Lab of Ornithology).

For this analysis, we included all HFBP calls regardless of whether a low frequency component was visible in spectrograms (i.e. HFBP-B), as this component was occasionally masked during noise events, making it difficult to consistently identify in spectrograms or audibly detect. The low-frequency component of HFBP-B calls has a median peak frequency of 1.5 kHz (Table 2), which overlaps with the dominant frequencies of vessel noise, contributing to its variable detectability. For the SLE call parameter analysis on Table 2, we selected only those produced during quiet periods when no vessel noise was present, ensuring that the low-frequency component of HFBP-B calls was audible and visible on the spectrograms. Thus, Table 2 presents the main acoustic parameters of 200 randomly selected good quality HFBP calls, including 100 HFBP-M (monophonic), and 100 HFBP-B (biphonal) calls. Fig. 5B illustrates both types of ultrasonic calls (HFBP-M and HFBP-B), as well as the broadband contact calls selected for this study.

While vessel noise could obscure portions of some vocalizations, most non-ultrasonic calls in our dataset were broadband signals, spanning a wide frequency range. This characteristic allowed us to detect at least the higher-frequency portions of these signals above the noise floor, even when lower-frequency components were masked. Similarly, for whistles, the fundamental frequency was sometimes obscured by noise, but harmonics remained visible in the spectrograms. In cases where certain components were masked, direct auditory detection was often challenging when listening to recordings with loud vessel noise. However, many of these calls could still be visually identified by carefully examining the spectrograms, where call structures were discernible behind darker, noise-dominated sections.

To ensure accuracy, all call classifications were conducted manually, as automated detection methods would struggle to reliably extract signals masked by vessel noise. This process involved systematically scrolling through each before/during/after event, adjusting contrast and saturation in the spectrograms, and modifying spectrogram parameters to enhance visibility. The manual component of this analysis was therefore critical in distinguishing vocalizations emitted during noisy periods and ensuring a robust dataset for comparison.

During each of the 21 events, the number of animals remained consistent across the before, during, and after periods, as we ensured that the entire herd was within 500 m. As our data are clustered (multiple measurements per noise event: before, during and after noise), we used the event ID (the 21 sequences before/during/after) as a random factor, which considers any potential between-events variance (e.g. due to number of animals or herd composition).

### Statistical analysis

We ran a Chi-square test to determine if the proportional use of ultrasonic, high frequency burst pulse calls (HFBP-M, HFBP-B) is independent of the population. We used R (R Core Team, 2022) and the glmer.nb function from the lme4 package (Bates et al., 2015) to perform generalized linear mixed models (GLMMs) with a negative binomial distribution, a log link function, and fit by maximum likelihood (Laplace approximation) to assess the relationship between call rates (number of calls/minute) and noise conditions (before, during, and after noise events). Noise treatment (before, during, after) and call type (CC, HFBP, and other) with interaction were included as fixed effects, while noise events were included as random effects. Model assumptions were checked using resDHARMa from the DHARMa package (Hartig, 2022). Overdispersion was examined using Ben Bolker's code (Bolker, 2024). Pseudo *R*^2^ values (variance explained by both fixed and random effects) for GLMMs were calculated using piecewiseSEM (Lefcheck, 2016) package according to Nakagawa et al. (2017). We did not include behavior as a variable. To obtain specific *P*-values for comparisons between noise conditions (before, during, after) across different call types, we used the estimated marginal means (emmeans) package (Lenth, 2024) to specify contrasts and applied Holm correction for multiple comparisons. This approach allowed us to directly compare noise conditions within the same model without rerunning it multiple times.
